# Supplementary material for: Surveillance for Metastasis in High-Risk Uveal Melanoma Patients: Standard versus Enhanced Protocols
Source: Cancers (Basel). 2023 Oct 17;15(20):5025. doi: 10.3390/cancers15205025 (PMC10605386; doi:10.3390/cancers15205025)
Supplement: Supplementary file 1 [file cancers-15-05025-s001.zip › Supplemental Table.pdf]

Supplemental Table. Metastasis risk modifiers by surveillance protocols

Feature	Standard Protocol vs Enhanced Protocol			Standard Protocol vs High Frequency			Standard Protocol vs Enhanced Modality		
	Standard Protocol (n = 11[1])	Enhanced Protocol (n= 76[1])	p-value[2]	Standard Protocol (n = 11[1])	High Frequency (n = 54[1])	p-value[2]	Standard Protocol (n = 11[1])	Enhanced Modality (n = 64[1])	p-value[2]
**Age (years)**	57 (50, 70)	63 (58, 70)	0.2	57 (50, 70)	63 (58, 69)	0.3	57 (50, 70)	64 (58, 70)	0.3
**Sex (Male/Female)**	7/4	37/39	0.4	7/4	27/27	0.4	7/4	30/34	0.3
**Tumor location**			0.3			0.2			0.3
Choroid only	10 (91%)	53 (70%)		10 (91%)	35 (65%)		10 (91%)	45 (70%)	
CBD +/- choroid	1 (9.1%)	23 (30%)		1 (9.1%)	19 (35%)		1 (9.1%)	19 (30%)	
**LBD (mm)**	13.0 (11.0, 15.8)	15.0 (11.0, 16.5)	0.6	13.0 (11.0, 15.8)	15.3 (11.3, 16.9)	0.5	13.0 (11.0, 15.8)	15.0 (11.0, 16.5)	0.6
**Tumor thickness (mm)**	6.7 (2.6, 10.0)	6.1 (3.8, 9.0)	0.9	6.70 (2.60, 9.95)	6.25 (4.00, 9.00)	0.9	6.7 (2.6, 10.0)	6.3 (3.7, 9.0)	0.9
**PRAME status (+)**	3 (60%)	21 (48%)	0.7	3 (60%)	19 (56%)	>0.9	3 (60%)	20 (50%)	>0.9

[1]Median (IQR); n (%)

[2]Wilcoxon rank sum test; Pearson's Chi-squared test; Fisher's exact test

CBD: Ciliary body; LBD: Largest basal diameter
