# Supplementary figures and images for: Surveillance for Metastasis in High-Risk Uveal Melanoma Patients: Standard versus Enhanced Protocols
Source: Cancers (Basel). 2023 Oct 17;15(20):5025. doi: 10.3390/cancers15205025 (PMC10605386; doi:10.3390/cancers15205025)

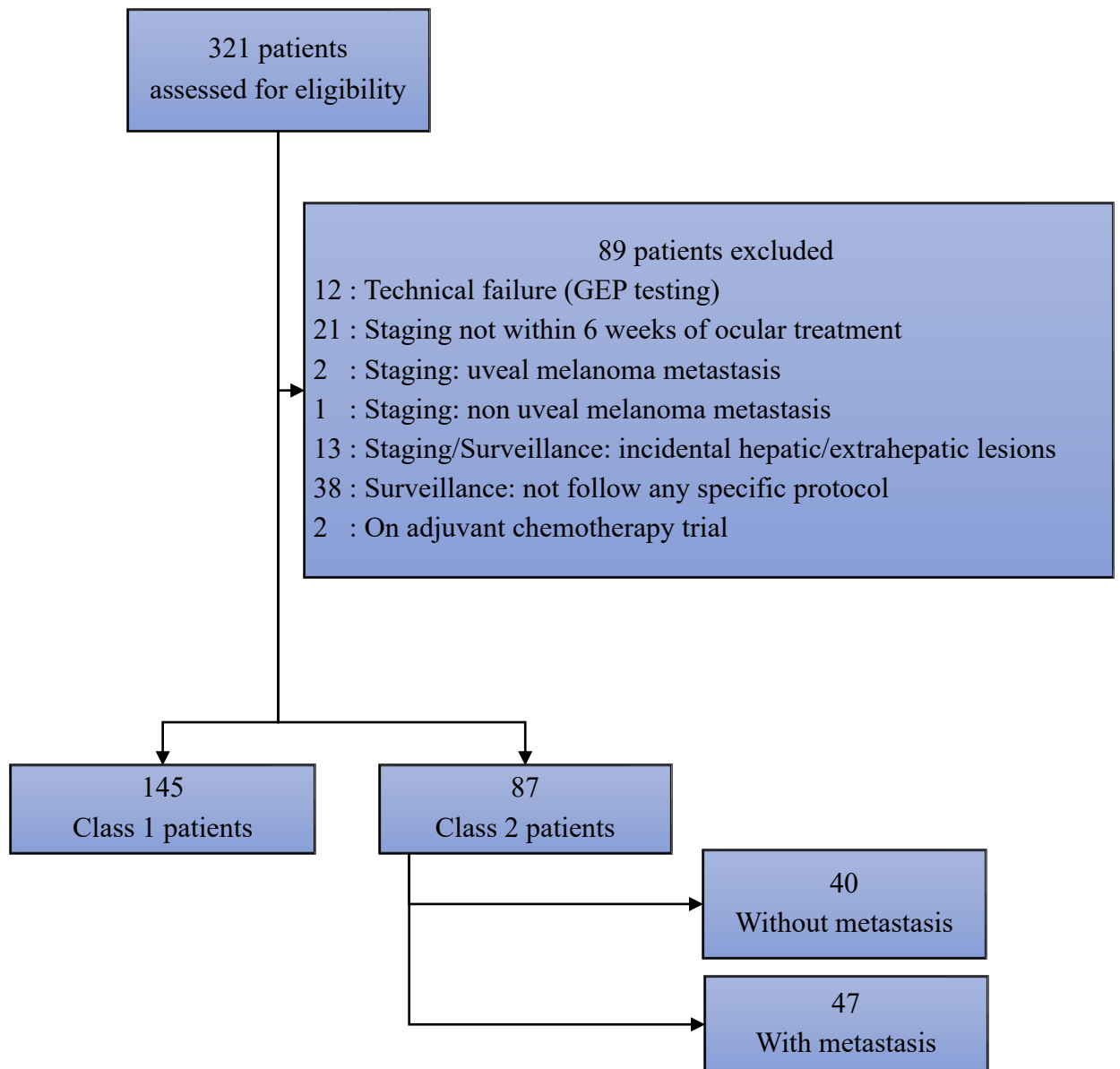

GEP: Gene expression profiling

Supplement: Supplementary file 1 [file cancers-15-05025-s001.zip › Supplemental figure.pdf]
